# Understanding decision-making for and against oncoplastic breast-conserving surgery as an alternative to a mastectomy in early breast cancer: UK ANTHEM qualitative study

**DOI:** 10.1093/bjs/znae133

**Published:** 2024-06-15

**Authors:** Charlotte Davies, Carmel Conefrey, Nicola Mills, Patricia Fairbrother, Chris Holcombe, Lisa Whisker, Joanna Skillman, Paul White, Douglas MacMillan, Charles Comins, William Hollingworth, Shelley Potter

**Affiliations:** Bristol Centre for Surgical Research, Population Health Sciences, Bristol Medical School, Bristol, UK; Population Health sciences, Bristol medical School, Bristol, UK; Population Health sciences, Bristol medical School, Bristol, UK; Independent Cancer Patients Voice (ICPV), Bristol, UK; Linda McCartney Centre, Royal Liverpool and Broadgreen University Hospital, Liverpool, UK; Nottingham Breast Institute, Nottingham University Hospitals NHS Trust, Nottingham, UK; Department of Plastic Surgery, University Hospitals Coventry and Warwickshire NHS Trust, Coventry, UK; Applied Statistics Group, University of the West of England, Bristol, UK; Nottingham Breast Institute, Nottingham University Hospitals NHS Trust, Nottingham, UK; University Hospitals Bristol and Weston NHS Foundation Trust, Bristol, UK; Population Health sciences, Bristol medical School, Bristol, UK; Bristol Surgical and Perioperative Care Complex Intervention Collaboration, Translational Health Sciences, Bristol Medical School, University of Bristol, Learning and Research Building, Southmead Hospital, Bristol, UK; Bristol Breast Care Centre, Southmead Hospital, Bristol, UK

## Abstract

**Background:**

Oncoplastic breast-conserving surgery may allow women with early breast cancer to avoid a mastectomy, but many women undergo more extensive surgery, even when breast-conserving options are offered. The aim of the ANTHEM qualitative study was to explore factors influencing women’s surgical decision-making for and against oncoplastic breast-conserving surgery.

**Methods:**

Semi-structured interviews were conducted with a purposive sample of women who had received either oncoplastic breast-conserving surgery or a mastectomy with or without immediate breast reconstruction to explore their rationale for procedure choice. Interviews were transcribed verbatim and analysed thematically. Trial registration number: ISRCTN18238549.

**Results:**

A total of 27 women from 12 centres were interviewed. Out of these, 12 had chosen oncoplastic breast-conserving surgery and 15 had chosen a mastectomy with or without immediate breast reconstruction. Overwhelmingly, women's decisions were guided by their surgical teams. Decision-making for and against oncoplastic breast-conserving surgery was influenced by three key inter-related factors: perceptions of oncological safety; the importance of maintaining/restoring femininity and body image; and practical issues. Oncological safety was paramount. Women who reported feeling reassured that oncoplastic breast-conserving surgery was oncologically safe were happy to choose this option. Those who were not reassured were more likely to opt for a mastectomy, as a perceived ‘safer’ option. Most women wished to maintain/restore femininity, with the offer of immediate breast reconstruction essential to make a mastectomy an acceptable option. Practical issues such as the perceived magnitude of the surgery were a lesser concern.

**Conclusion:**

Decision-making is complex and heavily influenced by the surgical team. High-quality, accurate information about surgical options, including appropriate reassurance about the short- and long-term oncological safety of oncoplastic breast-conserving surgery is vital if women are to make fully informed decisions.

## Teaser

Perceived overestimation of the oncological benefits of a mastectomy and a lack of awareness of the potential short- and long-term issues associated with breast reconstruction raise concerns about the quality of the information provided to women making decisions about their options and the degree to which their decisions are fully informed.

## Introduction

Breast cancer affects one in eight UK women, with approximately 55 200 new cases of breast cancer diagnosed each year^1^. Despite improvements in breast cancer treatment, up to 40% of women still undergo a mastectomy as their primary surgical treatment. Loss of a breast can adversely affect quality of life (QoL) and body image^2,3^, and, although the National Institute of Health and Care Excellence (NICE) recommends that all women having a mastectomy should be offered breast reconstruction to improve their well-being^4^, only 20–25% of women choose this option^5^. Furthermore, not all women may want or be considered suitable for reconstructive surgery due to patient- or cancer-related factors such as smoking or the need for post-mastectomy radiotherapy.

Oncoplastic breast-conserving surgery (OPBCS) describes a range of procedures that can largely be divided into volume displacement and replacement techniques that combine removing the cancer with plastic surgical techniques to lift, reduce, or partially reconstruct the breast. Both techniques allow larger volumes of tissue to be removed, while preserving excellent cosmetic outcomes, and may allow women not suitable for standard breast-conserving surgery to avoid a mastectomy^6^. OPBCS has in many studies been shown to be oncologically safe^7–10^ and results in significantly fewer complications^6^ and better patient-reported outcomes^11–14^ than mastectomy with or without immediate breast reconstruction (IBR). OPBCS has also been found to be more cost-effective compared with more extensive surgery^15–17^.

Given the significant benefits of OPBCS and the widespread availability of these procedures in the UK, as demonstrated in a recent national practice survey^18^, it may be expected that, if offered OPBCS, most women would choose oncoplastic breast conservation to avoid a mastectomy. However, the Getting it Right First Time initiative suggests that less than 10% of women in the UK currently have an oncoplastic procedure, whereas mastectomy rates may be as high as 50% in some centres^5^. The potential benefits of OPBCS for women with early breast cancer are therefore not being realized and it is unclear why women offered OPBCS as an alternative to a mastectomy choose more extensive surgery when this may be avoided. Understanding factors influencing decision-making in breast cancer surgery is vital to ensure that accurate information is provided to support women facing these decisions that are often challenging.

The ANTHEM study (ISRCTN18238549) explored the feasibility of undertaking a large-scale study comparing the clinical effectiveness and cost-effectiveness of OPBCS as an alternative to a mastectomy with or without IBR in women offered both options^19^. The qualitative phase of this study used semi-structured interviews to explore women’s perceptions of surgical choice and to understand factors influencing surgical decision-making for and against OPBCS.

## Methods

Full details of the ANTHEM study have been described elsewhere^19^. Full ethical approval was obtained (Wales Research Ethics Committee 6 Ref. No. 20/WA/0225) and the study was prospectively registered before commencing recruitment (ISRCTN18238549). The qualitative phase of ANTHEM has been reported according to consolidated criteria for reporting qualitative research (COREQ) guidelines^20^.

### Setting and participants

All women who consented to participate in the multicentre ANTHEM prospective cohort study were invited to express an interest in participating in the qualitative interview substudy. Full details are reported elsewhere, but, in brief, women were eligible to participate in the cohort study if they were aged 18 years and over and had been diagnosed with primary invasive breast cancer or ductal carcinoma *in situ* (DCIS) and were considered suitable for level 2 OPBCS with either volume replacement or displacement techniques as an alternative to a mastectomy with or without IBR by the multidisciplinary team. All participants were offered both OPBCS and a mastectomy with or without IBR as appropriate. Women underwent their procedure of choice and information regarding complications and patient-reported outcomes using the validated BREAST-Q questionnaire were collected at 3 and 12 months after surgery. These results will be reported elsewhere.

### Patient recruitment

Patients who expressed an interest in participating in the interview substudy were sent a participant information sheet, invitation letter with reply slip, and consent form by e-mail or post, depending on their preference.

### Sampling

Purposive sampling using a maximum-variation approach based on procedure choice (OPBCS using both volume replacement and displacement techniques, simple mastectomy, and a mastectomy with IBR using implants and autologous tissue techniques), age at diagnosis, and recruiting centre was used to recruit participants who returned completed reply slips. Theoretical sampling was used as the study progressed to include individuals whose views had the potential to enhance or disprove emerging themes. Sampling, data collection, and analysis were undertaken concurrently and iteratively until data saturation was reached and no new themes emerged from the data.

### Data collection

Telephone interviews were conducted using a semi-structured topic guide (*Supplementary material*) developed collaboratively by the ANTHEM steering group based on the literature and their specialist expertise. It explored women’s perceptions of surgical choice and their rationale for deciding for or against OPBCS and also included other areas of discussion such as expectations and experiences of their breast surgery. The topic guide was modified iteratively as the interviews progressed to allow developing themes to be explored.

Telephone interviews were conducted by an experienced female researcher (Charlotte Davies) who was independent of the patient clinical team. Interviews were conducted at a time convenient to the participant and written consent was obtained before each interview. All interviews were digitally audiorecorded and transcribed verbatim.

### Data analysis

Thematic analysis using the constant comparative approach of grounded theory^21^ was used to analyse the interview data^22^. Reflective notes were made after each interview to capture non-verbal cues and were taken into account in the analysis. Two transcripts were independently double coded by another experienced qualitative researcher (Carmel Conefrey) and codes discussed, agreed, and then applied to the data set.

## Results

A total of 202 women expressed an interest about taking part in the interview study of whom 88 purposefully selected individuals were invited to participate. Of these, 27 women replied and were interviewed. All interviews were undertaken over a 20-month interval from July 2021 to February 2023 and ranged in duration from 18 to 66 min (mean 35 min).

### Patient demographics

The 27 women were recruited from 12 UK ANTHEM centres. Details of study participants are summarized in *Table 1*. The median age was 55 (range 41–69) years and all were interviewed between 3 and 8 months after surgery. Most women participating in the ANTHEM cohort study chose OPBCS. Women electing to undergo a mastectomy with or without IBR were therefore specifically targeted to allow the views of this group to be fully explored. Overall, 12 women had OPBCS with volume replacement (5 women) or displacement (7 women) and 15 women chose a mastectomy with (10 women) or without (5 women) IBR; of the 10 women who underwent IBR, the procedure was implant based for 4 women and autologous for 6 women (*Table 1*).

**Table 1:** Demographics of the 27 women participating in the ANTHEM interview study

	OPBCS (TM) (*n* = 7)	OPBCS (LPF) (*n* = 5)	Mx only (*n* = 5)	Mx with IBR (autologous)* (*n* = 6)	Mx with IBR (implant based) (*n* = 4)	All women (*n* = 27)
**Age (years)**						
<45	2	1	0	1	0	4
45–60	4	3	3	5	3	18
>60	1	1	2	0	1	5
**Stage with**						
0	1	1	4	0	0	6
1	2	1	0	1	1	5
2	1	3	1	3	1	9
3	0	0	0	1	2	3
Not reported	3	0	0	1	0	4
**Treatments received**						
Chemotherapy†	1	1	1	5	2	10
Radiotherapy	6	5	3	4	2	20
Endocrine therapy	5	4	1	5	3	18
**Interview time point (months since surgery), *n***						
<6	5	3	5	3	1	17
≥6	2	2	0	3	3	10

Values are *n* (%) unless otherwise indicated. *One patient initially had a TM followed by a mastectomy with IBR (autologous). †Includes four participants who received neoadjuvant chemotherapy. OPBCS, oncoplastic breast-conserving surgery; TM, therapeutic mammaplasty; LPF, local perforator flap; Mx, mastectomy; IBR, immediate breast reconstruction.

### Perceptions of surgical choice

#### Theme 1: absence of meaningful choice

**Fig. 1:**
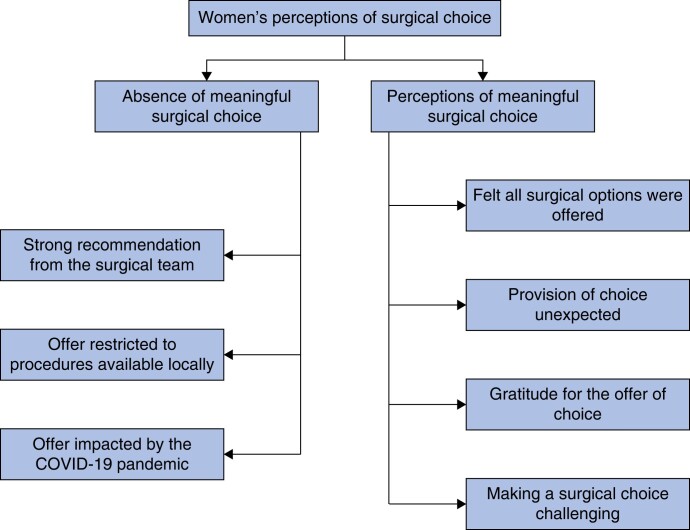
Thematic map of women’s perceptions of surgical choice, as identified from interviews

Although the offer of both OPBCS and a mastectomy was a prerequisite for study entry, not all women interviewed perceived that they were meaningfully offered a choice of surgical procedure. Three key subthemes were identified relating to the perceived lack of choice: a strong recommendation from the surgical team; offer restricted to procedures available locally; and offer impacted by the COVID-19 pandemic. The two themes regarding perceptions of surgical choice and their subthemes are summarized in *Fig. 1*, with illustrative quotes from participants available in *Table 2*.

**Table 2:** Themes and subthemes regarding women’s perceptions of surgical choice

Themes and subthemes	Example quotations
**Absence of meaningful choice**	
Strong recommendation from the surgical team	‘Then they got the biopsy results and they said “We’re recommending mastectomy”. Because it was a 4 cm tumour and it was in top centre part of the breast, a lumpectomy would have left quite a big dent. That was the rationale, I think.’ (Centre 3, Mx with IBR (autologous)) ‘I think he (the surgeon) was advising me more to have the mammoplasty; he felt it was more appropriate for me, I think.’ (Centre 3, OPBCS (TM)) ‘…But then I had an MRI scan and they discovered another very small tumour, I couldn’t even feel it. The surgeon said the breast was unstable really so he would be happier if it was all taken away. Then that’s when we discussed which kind of implant.’ (Centre 8, Mx with IBR (implant based)) ‘The way my biopsy and test results were presented to me and the case that the surgeon made for the mastectomy seemed quite cut and dry to me and quite clear. In a way, there was never really any particular discussion about an alternative…’ (Centre 6, Mx only)
Offer restricted to procedures available locally	‘They told me that the surgeon I had currently been under didn’t do implants at the time of surgery.’ (Centre 7, Mx with IBR (implant based)) ‘The surgeon I had; it was a DIEP she did. She didn’t offer the other ones.’ (Centre 12, Mx with IBR (autologous))
Offer impacted by the COVID-19 pandemic	‘I think a lot of it is probably to do with COVID, it just took me a long time to get in the system because of the delays…unfortunately by the time I got to surgery, what I had had grown so big it would’ve been life-threatening to have done anything other than a mastectomy.’ (Centre 1, Mx only)
**Perceptions of meaningful surgical choice**	
Felt all surgical options were offered	‘It was totally up to me, as I said. The initial discussion she went through every possible option of lumpectomy, mastectomy…. But yes, at the end of the day she said “It’s your choice. If you would like a mastectomy, I will certainly perform that. But here is another option for you”, which was the ICAP surgery, so all options were open to me.’ (Centre 2, OPBCS (LPF))
Provision of choice unexpected	‘I wasn’t really bothered about what kind of surgical procedure I had. If he’d (the surgeon) turned round and said that I needed a mastectomy, then that would have been absolutely fine…for me, it wasn’t about the cosmetic results. It was about getting rid of the tumour.’ (Centre 3, OPBCS (TM))
Gratitude for the offer of choice	‘So, I came out of that (patient consultation with surgeon) absolutely euphoric, to be quite honest with you. Because I went in, expecting that I was going to be losing my breast, and then came out that they were going to try to save the breast…and I thought that’s what we can do, that’s just brilliant…. There was an option to do something that was breast-conserving route rather than first response being immediate mastectomy…’ (Centre 4, OPBCS (TM))
Making a surgical choice challenging	‘I think when it comes down to it because you are all over the place, you kind of want them to just say “This is what you should do”, but they don’t…you have to choose what you want because you are the one that has to live with it.’ (Centre 11, Mx with IBR (implant based))

Mx, mastectomy; IBR, immediate breast reconstruction; OPBCS, oncoplastic breast-conserving surgery; TM, therapeutic mammaplasty; DIEP, deep inferior epigastric perforator flap; ICAP, inter-costal artery perforator flap; LPF, local perforator flap.

##### Strong recommendation from the surgical team

Many women perceived that they did not choose their procedure, but rather described how they felt that their surgical procedure had been recommended or advised by their surgical team. To these women, the justification of procedure provided by the surgical team seemed clear. They felt that it was obvious why this type of procedure was being offered and why it was the best option for them clinically. These participants reported being confident that their surgeon had recommended the best surgical procedure and did not question whether any other options may have been open to them. Strong trust in the surgeon was central to this acceptance and a key theme in this group (*Table 2*).

##### Offer restricted to procedures available locally

Some women described how the surgical options open to them were dependent on what techniques were available at their local hospital and the specific procedures their surgeon was trained to perform. Some women reported that the procedure they would have opted for was not performed at their local hospital or could not be performed by their surgeon. These women recalled feeling that they did not really have a meaningful choice, as not all the options were available to them (*Table 2*).

##### Offer impacted by the COVID-19 pandemic

A minority of women described that the COVID-19 pandemic and associated delays had impacted on the options available to them (*Table 2*).

#### Theme 2: perceptions of meaningful surgical choice

##### Felt all surgical options were offered

The majority of women reported feeling that they had been given a choice of procedure and offered all options by their surgeon. These women felt that they could make an informed choice on the type of breast surgery they wanted and could decide which procedure would be the right option for them.

##### Provision of choice unexpected

Several women described how choice was unexpected, as they had automatically assumed that they would need a mastectomy to treat their breast cancer. Many expressed a delight at being offered OPBCS as an alternative, but others relayed feeling disconcerted and surprised that they were asked to make a decision about what procedure they preferred. Some women reported that they did not have a strong preference and would have accepted any of the procedures offered to them.

##### Gratitude for the offer of choice

Many women felt pleased that they had been offered a choice and that they could actively participate in the decision-making process. These women reported that they were grateful that they were able to decide for themselves and have an element of control as to which procedure was best for them.

##### Making a surgical choice challenging

Although many women welcomed the opportunity to decide what procedure was right for them, some described feelings of considerable anxiety and uneasiness about having to make a choice and found the decision-making process difficult. A few women described how they would have preferred to have less choice, as they found trying to weigh up the risks and benefits of different options complex and emotionally challenging.

Some women perceived that choice was a burden and described feeling anxious about whether they were making the ‘right’ decision for their breast cancer treatment, knowing the condition was potentially life-threatening. They recalled that they were not in the best mental state to make such an important choice when they were still coming to terms with their breast cancer diagnosis. Some reported that they would have preferred the decision to have been made for them.

### Factors influencing decision-making for/against oncoplastic breast-conserving surgery

**Fig. 2:**
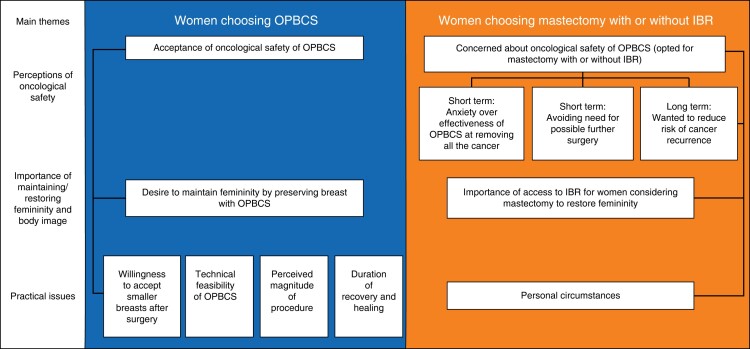
Thematic map of factors influencing women’s surgical decision-making for/against oncoplastic breast-conserving surgery, as identified from interviews OPBCS, oncoplastic breast-conserving surgery; IBR, immediate breast reconstruction.

Three main themes were identified as being central to women’s decision-making for and against OPBCS (*Fig. 2*).

#### Theme 1: perceptions of oncological safety

##### Acceptance of oncological safety was a prerequisite for acceptance of oncoplastic breast-conserving surgery

Women who reported feeling reassured that OPBCS was oncologically safe were happy to undergo the procedure. Oncological safety encompassed both short- and long-term concerns; short-term confidence that OPBCS was sufficient to remove all the cancer in the breast and long-term reassurance that choosing OPBCS did not lead to a higher chance of the cancer returning. Some women specifically commented that they had raised these issues with the surgical team. Women who reported feeling reassured by their surgical teams that OPBCS and a mastectomy had equivalent survival outcomes chose breast conservation. Those who did not universally chose a mastectomy (*Table 3*).

**Table 3:** Themes and subthemes regarding factors influencing women’s surgical decision-making

Themes and subthemes	Example quotations
**Perceptions of oncological safety**	
Acceptance of oncological safety of OPBCS	‘I did question the therapeutic mammaplasty. Will it rid my body of the cancer? What are the chances of it coming back? I would say that was more important than the cosmetic outcome…. If there was more chance of the cancer coming back by not having the breast off, I would’ve opted to have had the mastectomy.’ (Centre 5, OPBCS (TM)) ‘I guess the main thing with breast cancer patients and in my head was, well is a lumpectomy going to be enough to get rid of the cancer, does it reduce the risk of it coming back, would a mastectomy not be best? And I did ask that question in the clinic and was told, actually, the evidence is that lumpectomy is as good as mastectomy. And I felt quite reassured by that.’ (Centre 3, OPBCS (TM))
Concerned about oncological safety of OPBCS (opted for mastectomy with or without IBR)	‘There was no way we would have got it all.’ (in reference to the cancer if patient had opted for an OPBCS procedure) (Centre 6, Mx only) ‘I just right away when they said lumpectomy I said there’s no way. I don’t want to go through a lumpectomy and then be back 2 weeks later…. I kind of knew the lumpectomy was not going to be the solution for me. I knew I wanted reconstruction.’ (Centre 11, Mx with IBR (implant based)) ‘So I didn’t want to go for repeated surgeries in case the margin was not there, because that is the main reason why I decided to go for mastectomy.’ (Centre 12, Mx only) ‘I wasn’t even thinking of a mastectomy first of all, I was thinking of a lumpectomy. But then they were saying that my new surgeon said “Yes, we can. Your option is that”. He said “But, if the margin around the lump is not clear, we’ll have to go back and take more out, so that’s a second operation”. I just thought, I can’t do that. It’s got to be done all at once. If he (the surgeon) can do the implant at the same time, I’ll just do that.’ (Centre 7, Mx with IBR (implant based)) ‘No. I couldn’t do that (have an OPBCS procedure). I needed to know that it was all out. The most effective way is to take the whole breast away. I didn’t want that to happen and it affected my mental health terribly and still does 10 months later. But I didn’t see an option because I’m consumed with the cancer returning and I had to go with the best possible option for that not to happen.’ (Centre 7, Mx with IBR (implant based)) ‘I think I went for the better option (an Mx with IBR), the safer option. Because they said I didn’t want to constantly worry about the rest of the tissue in the boob…I just knew I had to get rid of the tissue in that boob because there was so much of it.’ (Centre 4, Mx with IBR (implant based))
Short-term concerns: Anxiety over effectiveness of OPBCS at removing all the cancer Avoiding need for possible further surgery Long-term concerns: Wanted to reduce risk of cancer recurrence	
**Importance of maintaining/restoring femininity and body image**	
Desire to maintain femininity by preserving breast with OPBCS	‘I think I would have felt that there was something missing (having an Mx) and possibly, you know, when you read all the other questions about how feminine do you feel. I think you would lose a little bit of that because you only then have one breast instead of two.’ (Centre 2, BCS (LPF)) ‘…But I think my thoughts were, well, if I can keep the breast then I would like to try and go down that route…I just felt I would rather try and keep what I have, if that makes sense, it’s difficult to explain.’ (Centre 2, OPBCS (LPF))
Importance of access to IBR for women considering mastectomy to restore femininity	‘I think for myself I wanted to come away feeling feminine. I wanted a feminine outlook because I really thought it would be destructive to my well-being…because one option is you could just have nothing there at all and they’d remove the entire breast. I said right from the start I do not want that either, I want to maintain having a female form…’ (Centre 11, Mx with IBR (implant based)) ‘So having this option where you go to sleep knowing your boob is going to be removed but you know when you wake up you will have a boob, just not the same one, it actually psychologically helps massively…. But I think to have it removed (your breast), to know you need a mastectomy is horrific, but to know you’ll have a reconstruction straightaway so you won’t see the results of the mastectomy, if that makes sense? You won’t have this flatness actually really really makes a difference.’ (Centre 12, Mx with IBR (autologous)) ‘If you lose that (your breast), it’s some of what makes you a girl…. If I had gone flat, I think it would have been a much bigger impact mentally, on me as a girl. Feeling less feminine, less attractive than what I’ve got. I do not regret at all, the extra surgery and the extra healing time.’ (Centre 3, Mx with IBR (autologous))
**Practical issues**	
Willingness to accept smaller breasts after surgery	‘…I had quite large breasts for my age, and they were just getting worse, and I was really unhappy. So actually, for me, it was great, because I thought this is fab, I can get these reduced and put back to shape. So, I went for that.’ (Centre 3, OPBCS (TM))
Technical feasibility of OPBCS	‘I was expecting him (the surgeon) to probably say to me that that would be what I would be having done (a Mx). And to his great credit, he said “Well I need to examine you first”. Because he said “The way we go with this ultimately going to depend on many issues, one of which is ultimately going to be the size of your breast” and I have 36D breasts. So, I said “oh that’s news to my ears, that’s fantastic” because I’ve got very large breasts…. “I can’t understand why you would automatically give me a mastectomy. Because there’s an awful lot of breast here”.’ (Centre 4, OPBCS (TM))
Perceived magnitude of procedure (OPBCS less invasive/radical than mastectomy with or without IBR)	‘Therefore, out of the two choices (Mx or OPBCS) I thought let’s go for the less invasive procedure and the better outcome, you know cosmetically.’ (Centre 2, OPBCS (LPF))
Duration of recovery and healing	‘He (the surgeon) didn’t feel that (a Mx) was necessary. And also, I figured just surgery on one side rather than two would be easier for possible infection, healing, recovery, all those sorts of things.’ (Centre 10, OPBCS (LPF))
Personal circumstances	‘It seemed to me like it was the best thing to do and that I just had to woman up and get on with it. Actually, I don’t know whether it’s to do with my age…I’m post-menopausal so I’m not a young woman looking to have children and I’m not in a relationship. I didn’t struggle with the decision.’ (Centre 6, Mx only) ‘I knew straightaway that I needed a mastectomy, but my husband had a brain tumour removed in 2013, so I’ve been his carer. Plus, the location, where we live, it’s pretty rural.’ (Centre 6, Mx only) ‘…at my age, I didn’t feel reconstruction was necessary…the time involved in recovery. Plus, reconstruction never came into my mind anyway…knowing the possibilities of infections and sepsis and all the other complications that could arise.’ (Centre 6, Mx only)

OPBCS, oncoplastic breast-conserving surgery; TM, therapeutic mammaplasty; Mx, mastectomy; IBR, immediate breast reconstruction; LPF, local perforator flap.

##### Mastectomy was chosen when oncoplastic breast-conserving surgery was not felt oncologically safe

For many women, a mastectomy was perceived as the safer option, with both short- and long-term concerns about the oncological safety of OPBCS identified as key drivers for decision-making. Women felt they would be more in control of the cancer by having a mastectomy and would have a higher chance of cure (*Fig. 2* and *Table 3*).

##### Anxiety over effectiveness of oncoplastic breast-conserving surgery at removing all cancer and avoiding further surgery

Several women expressed concerns about whether OPBCS would remove all the cancer from the breast. Many described their anxiety about the possibility of further surgery for involved or positive margins after OPBCS. The potential of further surgery was a real concern for some women and something they were not willing to risk. They described not wanting to put themselves through more operations than was necessary and expressed a strong desire for their surgery ‘to be done all at once’. Women wanted to know that there was absolutely no possibility of any cancer remaining and they described having more confidence that a mastectomy would give them that reassurance and peace of mind. Many women described having strong feelings about their choice to have a mastectomy and, for some, it was an almost instant preference and knowing that they would not change their minds (*Table 3*).

##### Reducing the risk of recurrence

Many women expressed a fear of cancer recurrence and reported that they would rather ‘get rid of it all’ and ‘take it all off’ than opt to have OPBCS. These women described their anxiety about their cancer coming back and the possibility of it spreading in the future. These women perceived that a mastectomy procedure reduced the risk of their cancer returning, thus minimizing their stress and worry, and giving them a better long-term outcome.

#### Theme 2: importance of maintaining/restoring femininity and body image

##### Desire to maintain femininity by preserving breast with oncoplastic breast-conserving surgery

Many women reported concerns about losing their breast and expressed a desire to be able to keep as much of the breast tissue as possible. They described how they felt this was a better option for them surgically in terms of feeling ‘normal’ and knowing their breast/breasts was/were ‘still their own’. Other women reported that preserving their breast made them feel ‘not quite so different’ and were pleased to be able to maintain their femininity. The thought of losing their breast was often distressing and several women described feeling overjoyed when OPBCS was offered as an alternative to a mastectomy.

##### Importance of access to immediate breast reconstruction for women considering mastectomy

For many women, a mastectomy was only an acceptable option if IBR could be performed to restore femininity. These women described how they had not wanted to be flat chested after a mastectomy and expressed concerns about how this would have looked. They were also concerned that a simple mastectomy without reconstruction would have had a negative effect on their mental health and described that they would have struggled with the idea of waking up after surgery to find their breast/breasts removed (*Table 3*).

Many women described how important an IBR was as a means of restoring a female form and in terms of feeling feminine and attractive. One woman explained that to lose a breast completely would be like losing ‘what makes you female’. Concern over appearance and knowing that a reconstruction could be done at the same time as a mastectomy were important factors for many women. Indeed, it was only the availability of IBR that made a mastectomy an acceptable option for many women.

#### Theme 3: practical issues

Although oncological safety and body image were the main drivers of women’s surgical decision-making, surgery choices were also influenced by practical issues. Practical considerations were raised for all surgical options. See *Table 3*.

##### Willingness to accept smaller breasts after surgery and technical feasibility of oncoplastic breast-conserving surgery

Some women described that they had been happy to choose OPBCS, as they were keen to change and reduce their breast size. These women tended to have larger breasts and they were delighted to be able to have them reduced and reshaped at the same time as having the cancer removed. The option of having a reduction at the same time as removing the cancer was perceived as an additional benefit and positive reason for having chosen an OPBCS procedure such as a therapeutic mammaplasty (TM).

##### Perceived magnitude of procedure and duration of recovery

Some women perceived that having a mastectomy was a far more radical and extensive surgical procedure than OPBCS. These women opted for OPBCS, as they perceived that it led to a quicker recovery, with fewer complications and less time in hospital.

##### Personal circumstances

Other women described that their choice to have a mastectomy was due to personal circumstances, including where they lived, their age, not being in a personal relationship, and having caring responsibilities at home. They perceived that a simple mastectomy was the quickest way to complete treatment and avoid any potential further complications.

## Discussion

This study used qualitative methods to explore factors influencing women’s decision-making for and against OPBCS. Despite the offer of both OPBCS and a mastectomy being a prerequisite for study entry, not all women perceived that they were offered meaningful choice. This was often because only limited options were discussed, sometimes because they could not be offered locally. Several women described receiving strong treatment recommendations from their surgical team, which was often accepted without question, highlighting the central role of the surgeon in the decision-making process. Many women described how they expected to be told they needed a mastectomy, but were delighted to be offered the opportunity to avoid such extensive and life-changing surgery. Most women felt very happy to be involved in choosing the type of surgery that they considered was right for them, but some described feeling under pressure to make the ‘right’ decision and found the offer of choice stressful and challenging. When choice was given, perceptions of oncological safety were pivotal to women’s acceptance of OPBCS, with those concerned about incomplete cancer excision, concerned about the need for further operations, or worried about longer-term recurrence risk opting for a mastectomy as the perceived ‘safer’ option. Many women desired either to preserve their femininity by choosing oncoplastic breast conservation or to restore a feminine form by opting for IBR. A mastectomy was only acceptable to many women when combined with reconstruction, as several women described the idea of ‘being flat’ as extremely distressing. Practical issues were a lesser concern for many women.

This study highlights the profound impact that surgical teams have on women’s decision-making. It is therefore vital that surgeons are able to provide accurate, balanced, and up-to-date information about the risks and benefits of all available options. Women in the study repeatedly referred to a mastectomy as a ‘safer’ option and, although concerns about the need for further surgery due to margin involvement are valid, OPBCS is associated with a lower risk of re-excision than standard breast-conserving surgery^23^. However, the perception that a mastectomy reduces the risk of recurrence and improves breast cancer survival may be misplaced, as several recent meta-analyses have shown a survival benefit for breast conservation over mastectomy^24–26^. Caution is needed extrapolating these findings to OPBCS, but current evidence suggests long-term oncological outcomes are at least equivalent to a mastectomy^7–9,23^. Breast reconstruction was perceived by many as a panacea after a mastectomy; a direct replacement for the natural breast that would restore their femininity and allow them to feel ‘normal’ again. None of the participants reported that their surgical teams had discussed the limitations of breast reconstruction such as the need for revisional surgery or the long-term outcomes. This highlights that women were not making fully informed decisions about reconstructive surgery. The complication rates for reconstruction are much higher than those for OPBCS^6,27^. Moreover, the long-term outcomes can be poor, particularly after implant-based procedures, which are associated with high numbers of revisions^28^ and inferior patient-reported outcomes^29^. Perceived overestimation of the oncological benefits of mastectomy and a lack of awareness of the potential short- and long-term issues associated with breast reconstruction raise concerns about the quality of the information provided to women making decisions about their options and the degree to which their decisions are fully informed. Interestingly, concerns regarding oncological safety were largely not related to their stage of disease and this did not appear to be a factor influencing their decision-making. This highlights the need for better information to support shared decision-making and this has recently been identified as a key research priority^30^.

This study is, to the authors knowledge, the first to use qualitative methods to explore, in depth, women’s decision-making for and against OPBCS as an alternative to a mastectomy for treatment of early breast cancer. The results highlight some key issues, but there are limitations of this work that need consideration. First, this is a qualitative study involving 27 women and it is possible that the views of this cohort may not be representative of women more broadly. However, participants were purposively sampled based on procedure type and age from 12 diverse UK breast units of differing size and geographical location. The views presented here are therefore likely to be reflective of experiences of women more broadly. Participants were asked to recall the decision-making process from an emotionally challenging time when they were facing a new breast cancer diagnosis. It is therefore possible that they were unable to accurately recall what information had been provided by their surgical teams or the factors that impacted their decision. It is also possible that they misinterpreted aspects of the information provided. Audiorecording of consultations would provide a complimentary strategy to explore the decision-making process and may be a potential area for future work. However, women in this study have provided valuable insights into their interpretation of the information provided, which is perhaps most relevant, as this interpretation would have been the basis for the decision that they made. Recall bias is possible, but breast cancer surgery is a highly salient event in a women’s life, so this is unlikely and, as interviews were conducted between 3 and 8 months after surgery, the risk of bias was minimized. Despite these limitations, the present findings provide a unique insight into women’s perceptions of surgical choice and factors influencing surgical decision-making.

This study highlights the urgent need for surgical teams to provide clear, accurate, and balanced information about the risks and benefits of all available surgical options to support women making decisions about surgery for early breast cancer. It suggests that when women are given high-quality information, including accurate information about the short- and long-term oncological outcomes of OPBCS, most are very happy to choose this option and avoid a mastectomy. Work is now needed to explore what information women want about different options and how this is best shared to support informed decision-making. Decision aids for breast-conserving surgery *versus* mastectomy^31^ and breast reconstruction^32,33^ have previously been developed, but have not been widely implemented into practice. Reasons for this are unclear, but alternative, more flexible strategies that can be tailored to individual patients are likely to be needed in the future and co-development of these strategies with patient advocates may be the most effective way by which these may be conceptualized and created. A further concern highlighted here was inequality regarding access to appropriate surgical options at participating centres. Inequality regarding access to breast reconstruction has been demonstrated previously^34^, but equitable access to options that may allow women to avoid a mastectomy and therefore the need for breast reconstruction is equally, if not more, important. Work is now needed to explore how this can be addressed, so that all women with breast cancer have equitable access to all options and the information and support necessary to allow them to decide what option may be right for them.

## Supplementary Material

znae133_Supplementary_Data

## Data Availability

The data set (transcripts) have not been uploaded to a public data repository owing to privacy concerns, but the authors will be able to consider specific requests on an individual basis.
