# Supplementary material for: Understanding decision-making for and against oncoplastic breast-conserving surgery as an alternative to a mastectomy in early breast cancer: UK ANTHEM qualitative study
Source: Br J Surg. 2024 Jun 15;111(6):znae133. doi: 10.1093/bjs/znae133 (PMC11179107; doi:10.1093/bjs/znae133)
Supplement: znae133_Supplementary_Data [file znae133_supplementary_data.docx]

Understanding women’s decision making for and against oncoplastic breast conserving surgery as an alternative to mastectomy in early breast cancer: UK ANTHEM qualitative study

Charlotte Davies^1^, Carmel Conefrey^1^, Nicola Mills^1^, Patricia Fairbrother^7^, Chris Holcombe^2^, Lisa Whisker^4^, Joanna Skillman^3^, Paul White^5^, Douglas MacMillan^4^, Charles Comins^6^, William Hollingworth^1^ and Shelley Potter^8,9^

^1^Bristol Centre for Surgical Research, Population Health Sciences, Bristol Medical School, Canynge Hall, 39 Whatley Road, Bristol, BS8 2PS, UK, ^2^Linda McCartney Centre, Royal Liverpool and Broadgreen University Hospital, Prescot Street, Liverpool, L7 8XP, UK, ^3^Department of Plastic Surgery, University Hospitals Coventry and Warwickshire NHS Trust, Clifford Bridge Road, Coventry, CV2 2DX, UK, ^4^Nottingham Breast Institute, Nottingham University Hospitals NHS Trust, Hucknall Road, Nottingham, NG5 1PB, UK, ^5^Applied Statistics Group, University of the West of England, Bristol, UK, ^6^University Hospitals Bristol Foundation NHS Trust, ^7^Independent Cancer Patients Voice (ICPV) and ^8^Bristol Surgical and Perioperative Care Complex Intervention Collaboration, Translational Health Sciences, Bristol Medical School, University of Bristol, Learning and Research Building, Southmead Hospital, Southmead Road, Bristol, BS10 5NB,UK ^9^Bristol Breast Care Centre, Southmead Hospital, Southmead Road, Westbury-on-Trym, Bristol, BS10 5NB, UK.

Corresponding author: Charlotte Davies, Bristol Centre for Surgical Research, Population Health Sciences, Bristol Medical School, Canynge Hall, 39 Whatley Road, Bristol, BS8 2PS, UK

Email: Charlotte.Davies@bristol.ac.uk . ORCID ID: 0000-0002-3468-4258

Supplementary material

Semi-structured interview schedule for the ANTHEM Study

A telephone interview will take place at a time convenient to the patient being interviewed.

# Introduction

- Introduce self and research questions (the researcher conducting the interview will also let the patient know that she is independent from the medical team and is not medically trained).
- Explain need to record interview and test audio recorder
- Written consent obtained prior to interview*– verbally confirm consent with patient to interview and audio recording. (*patient completes and returns an interview consent form after consenting to be contacted about the interview study on the ANTHEM prospective cohort study consent form)

# Research questions

1. What surgical option (oncoplastic breast conservation or mastectomy +/- immediate breast reconstruction) did the patient chose and why? What information did they need to help them make their decision?
2. What are the patient’s perceptions of the outcomes of surgery; did it meet their expectations?
3. What do they feel are the most important outcomes to be measured in a future research study comparing different types of surgery
4. What are their views of the PROMs we are planning to use (BREAST-Q scales; ICECAP-A and EQ-5D-5L)

**Background information and clarification of details**:

- Full name*,
- Date of birth*,
- Unit where reconstruction was performed*
- Date of surgery*
- (*This information will be separately noted and checked by the interviewer with the patient verbally over the phone before any audio recording of the interview begins)
- Clarify type of surgery
  - Oncoplastic breast conservation
    - Therapeutic mammaplasty
    - Local perforator flap
  - Mastectomy only
  - Mastectomy and immediate reconstruction
    - Using implants
    - Using their own tissue
- How did your surgery go? (opening question to begin interview)
  - Did you need any further surgery or a return to hospital?
- Did you have any treatment following your surgery such as chemotherapy, radiotherapy or hormone treatment?
  - If so, are you still having treatment or has this finished?

**Decision-making for surgery**

*We would like to understand surgery choices that women make and why women choose different types of surgery?*

*What influences women’s decision making?*

*What information is helpful for decision making?*

**Introduction:**

We are interested in understanding why women chose specific types of surgery and what information is needed to help them decide.

- You had a [insert procedure confirmed above]

*Would you be able to tell me what procedure you had ?(pause for answer) what would you say was the main reason you choose that procedure ?*

- - *What other procedures were you offered?*
  - *Probe why this procedure was recommended – what did surgeon suggest were the risks/benefits*
  - *Was there anything in particular that influenced your decision making?*
  - *What were your thoughts about the procedure/ procedures that were offered to you ?*
  - *Why did you choose [insert procedure]?*
  - *Did you need any treatment following your surgery (chemo, RT , hormone Tx)?*
  - *Did you need any radiotherapy ? Did this influence your decision making regarding the procedure or where you would have to travel to receive RT?*

*What were your thoughts/concerns about the different procedures that were offered to you?*

*Any longer term reasons for your decision regarding the procedure you had (length of recovery time/time in hospital/further treatment etc)*

*Those having Mx: Any reason why didn’t want to have breast conserving surgery, worried about how it would look? Worried about cancer coming back?*

*Those that had OPBCS: Any reason why you didn’t want to have a mastectomy? Concerns about how it would look?*

- *What did you take into account in making your decision?(where does the weight lie*)

***Probes*** – complications related to a specific procedure;

potential benefits;

need for adjuvant treatment following surgery ;

other medical problems;

social factors eg smoking,

what your family and friends said, what your surgeon said, what your breast nurse specialist said)

***Information provision***

- - *What information were you given about the different procedures? (e.g. written, photos etc)*
  - *Were you given booklets/ books, photographs ?(names of books/booklets useful)*
  - *Did you find online info useful/online forums ?(which websites, how did you find these? were you given websites by the team?)*
  - *What sort of things did the forumss talk about?*
  - *Who gave you the written information? (nurse, surgeon, macmillan support?)*
  - *What information was important to you in making your decision?*
  - *What /any other information would be helpful for decision making?*
  - *The amount of information given adequate?*
  - *Did you feel you were given enough information to make an informed choice about the surgery?*
  - *Having had surgery, is there any other information, that with hindsight, you would have liked to have had when making your decision?*

**Expectations of surgery and outcomes** (We are interested to know women’s expectations of surgery outcomes)

- *What were your expectations of the procedure you had ?*
- *How do the results of your surgery compare to your expectations?*
- *Were these expectations met?*
- *Anything you wish you had been told?*
- *What were you expecting would be the outcome of (name procedure)?*

*Probes:*

*satisfaction with breasts,*

*cosmetic look,*

*physical /psychosocial well-being,*

*recovery time,*

*pain in the breast area or when lifting / moving arms,*

*quality of life,*

*sexual well-being?*

- *Thinking about cosmetic look, physical movement, emotional wellbeing, satisfaction with breasts etc. (the outcomes)*
- *Which of these outcomes are most important to you? (can explore before and after surgery)*
- *Are you happy with the outcome of your breast surgery? (take one outcome theme at a time)*
- *Are you happy with how it feels and looks?*
- *Looking back, do you feel that your expectations of the procedure/recovery/outcome* were met?
  - *How satisfied are you with your breasts now?*
  - *How are you feeling emotionally about your breast surgery/reconstruction? Are you able to do what you want to do physically? If not, why not?*
  - *If no – are you able to tell me what you can’t do physically at the moment?*

**Overall/Reflection of their procedure:**

- *Were your expectations of surgery fulfilled?*
- *If could make the decision again, would you make the same one? If not, why not?*
- *What advice would you give to other women in a similar position, what would you tell them?*
- *What would you tell women who are considering having the same type of surgery*
- *Would you recommend the surgery you had?*
- *How would you make sure they felt prepared?*
- *Any other comments about your breast surgery? (Anything particularly good or could be improved?)*

**Views of future study design** (We are planning a larger study comparing different types of surgery in the future and want to know what the most important areas/factors are to measure, so these are cosmetic satisfaction, physical, mental wellbeing)

- *Which factors/outcomes do you think would be the most important to measure in a future research study comparing different types of surgery? (cosmetic Satisfaction with breasts, physical/mental well-being, cosmetic outcome etc?)*
- *Any that are more important than the other you would say?*
- *Are they equally important?*

*(So at the moment for this feasibility study we are asking women to complete post op questionnaires at 3 and 12 month after their date of surgery*

- *Do you think the timing of the questionnaires (at 3 and 12 months after surgery) are good time points to measure outcomes?*
- *How do you feel about these time-points, are they good points in time to reflect on the impact of surgery? if not, what time would be better?*

**Views on ANTHEM study questionnaires (BREASTQ, EQ-5D, ICECAP-A)** (Just wanted to ask you about your thoughts of the questionnaires you have completed as part of the main study , these will provide important information about how women feel about the outcomes of their surgery- and whether the questionnaires we are using appropriately cover issues that are important to women having breast surgery)

Interviewer will remind patient about the study questionnaires she has completed for the main ANTHEM cohort study. A sample questionnaire can be sent to the patient prior to the interview so the patient can be reminded about the content of the questionnaire.

- *Do you feel the questions covered in the questionnaires are relevant and adequate?*
- *Do they address issues that are important to you?*
- *Is there anything that you felt was missed?(that are important to ask?)*
- *Any questions which aren’t relevant?*
- *In particular, questions asked about each outcome (e.g satisfaction with breasts, emotional/physical well-being, quality of life etc) did they adequately (are they appropriate/ acceptable) capture issues that are important/relevant to you?*
- *Which were the most important areas do you think?*
- *Are there any questions you think should be asked that have not been covered in the questionnaire/any missing key concerns?*
- *Did the standalone questions/individual items in the questionnaire reflect issues important to you? Were the items acceptable/adequate?*

**Closing the interview**

- Thank them for their time
- Any final comments they would like to make?
